# Investigating the relationship between COVID-19-related and distress and ICD-11 adjustment disorder: two cross-sectional studies

**DOI:** 10.1192/bjo.2020.158

**Published:** 2020-12-28

**Authors:** Menachem Ben-Ezra, Wai Kai Hou, Robin Goodwin

**Affiliations:** School of Social Work, Ariel University, Israel; Department of Psychology, Centre for Psychosocial Health, The Education University of Hong Kong, Hong Kong, China; Department of Psychology, University of Warwick, UK

**Keywords:** COVID-19, serious mental illness, ICD-11, adjustment disorder, health

## Abstract

**Background:**

To assess the prevalence of elevated risk of serious mental illness and probable ICD-11 adjustment disorder in the UK population at two time points during COVID-19, and their association with COVID-19-related stressful events.

**Aims:**

To check the dose–response model for stress between the number of COVID-19-related stressful events and mental health indices.

**Method:**

We conducted two cross-sectional studies, using internet survey samples across the UK (*N* = 1293 for study 1; *N* = 1073 for study 2). Samples used internet panel surveys during March–April 2020 and 3 months later (June 2020), and used random stratified samples. Studies assessed prevalence of serious risk of mental illness and probable ICD-11 adjustment disorder.

**Results:**

Elevated risk of serious mental illness was found among those with COVID-19-related social life or occupationally stressful events (study 1). Elevated risk of serious mental illness and probable ICD-11 adjustment disorder was evident among those reporting COVID-19-related stressful events (personal health problems and caregiving; study 2). Cumulative COVID-19-related stressful events were associated with elevated risk of serious mental illness in study 1 (odds ratio 1.65; 95% CI 1.03–2.64; *P* = 0.037), and with both elevated risk of serious mental illness (odds ratio 2.19; 95% CI 1.15–4.15; *P* = 0.017) and probable ICD-11 adjustment disorder (odds ratio 2.45; 95% CI 1.27–4.72; *P* = 0.007) in study 2.

**Conclusions:**

Psychiatrists should be aware that COVID-19-related stressful events can lead to serious psychological problems. Mental health professionals need to pay particular attention to patients who report cumulative COVID-19-related stressful events, and consider them for mental health assessment and treatment.

SARS-CoV-2, the virus that causes COVID-19, first emerged in the UK in January 2020.^1^ The UK government closed schools on 20 March 2020, and imposed a nationwide lockdown on 23 March 2020.^2^ This lockdown immediately generated COVID-19-related stressful events, including stressors that were social (restricted face-to-face interactions, including with intimate and familial relations), occupational (unemployment, furlough, reduced hours, working from home) and educational (cancelled courses, changed schedules, remote studying). In addition, infection outbreaks led to a number of individuals being placed in quarantine. A recent Chinese study showed that quarantine restricted every aspect of life and increased mental burden.^3^ Although isolation restricts freedom of movement, other restrictions in COVID-19-related stressful events may stem from different sources. According to the conservation of resources theory^4^ and related frameworks,^5^ these major disruptions in daily lives can provoke psychological distress. During the COVID-19 pandemic, individuals may experience loss of those resources normally used to effectively cope with, and manage stressful situations. We suggest that this threat is cumulative across COVID-19-related stressful events, with the overwhelming effect a psychological version of a ‘cytokine storm’, an immune system failure with serious psychological implications.

We examine associations among COVID-19-related stressful events, underlying health conditions associated with increased mortality from COVID-19, being in isolation and serious mental illness. In addition, we hypothesise that a cumulative loss of resources will be associated with elevated risk of serious mental illness, with the larger the number of events generated by COVID-19, the greater the mental toll. We collect national cross-sectional data at two time points: the first survey during the peak of the pandemic (average new daily cases 3206, with 2078 new deaths), and the second data wave as the pandemic plateaued (average new daily cases 1042, with 137 new deaths; Fig. 1). Understanding the differential effects of disruption caused by COVID-19 during two very different periods should provide clinicians with a better understanding of its negative effect on mental health outcomes over the life course of the pandemic.

**Fig. 1:**
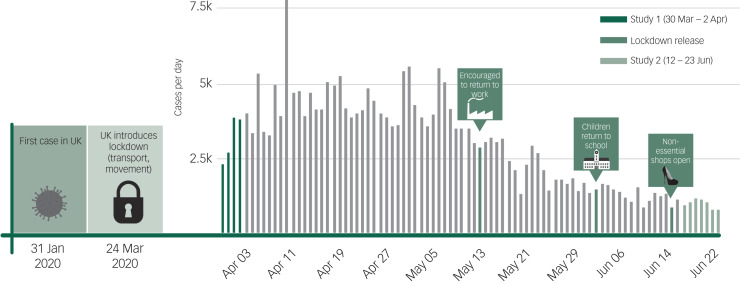
Data collection against UK pandemic timeline. Infographic was provided by David Goodwin based on figures from https://www.worldometers.info/.

## Method

All procedures performed in studies involving human participants were in accordance with the ethical standards of the Ethics Committee of Ariel University (approval number AU-SOC-MBE-20200225), and with the 1964 Helsinki Declaration and its later amendments or comparable ethical standards. Each participant signed an electronic informed consent form.

### Study 1

#### Sampling

We conducted a sample of the UK population by using an internet panel (*N* = 1293), with a random and stratified sampling. Participants were recruited between 30 March and 2 April 2020 (i.e. 1 week after the UK national lockdown). The mean age of the participants was 51.51 years (s.d. 14.75, range 18–75), 53.3% were female (*n* = 689), 69.0% reported being in a committed relationship (*n* = 892) and 27.2% (*n* = 352) reported having a background medical condition (hypertension, diabetes, cardiovascular disease, chronic respiratory disease, chronic obstructive pulmonary disease and cancer). The response rate was 37%. See Table 1 for more information.

**Table 1:** Descriptive statistics in the UK samples from March–April 2020 (study 1; *n* = 1293) and June 2020 (study 2; *n* = 1073)

Variables	Study 1				Study 2			
	Mean	s.d.	*N*	%	Mean	s.d.	*N*	%
Demographics								
Age, years	51.51	14.75			54.36	12.14		
Gender, female			689	53.3			591	55.1
Being in a committed relationship, yes			892	69.0			757	70.5
Being in risk group for COVID-19, yes			352	27.2			307	28.6
Lockdown								
Currently in isolation, yes			389	30.1			124	11.6
Disrupted COVID-19-related stressful events								
High disruption in social domain, yes			547	42.3				
High disruption in occupational domain, yes			442	34.2				
High disruption in educational domain, yes			485	37.5				
Index of disrupted COVID-19-related stressful events								
No disruption in COVID-19-related stressful events^a^			513	39.7				
One disruption in COVID-19-related stressful events			315	24.4				
Two disruptions in COVID-19-related stressful events			236	18.3				
Three disruptions in COVID-19-related stressful events			229	17.7				
COVID-19-related stressful events								
Financial problems, yes							217	20.2
Work problems, yes							177	16.5
Educational problems, yes							41	3.8
Housing problems, yes							61	5.7
Relationship problems, yes							140	13.0
Personal health problems, yes							321	29.9
A loved one's health problems, yes							303	28.8
Caregiving problems, yes							190	17.7
Index of COVID-19-related stressful events								
No events^a^							486	45.3
One event							193	18.0
Two events							170	15.8
Three events							101	9.4
Four or more events							123	11.5
Outcome variables								
Elevated serious mental illness (K6 ≥ 13)			215	16.6			153	14.3
Probable ICD-11 adjustment disorder^a^							171	15.9

a Original algorithm as proposed by Shevlin et al.^7^

#### Measurements

Being in isolation was measured by the item: ‘Are you currently in quarantine because of the coronavirus?’ (0 for no, 1 for yes).

COVID-19-related stressful events were measured by three questions: ‘In the past month, has the COVID-19 pandemic affected your relationships or social life?’, ‘In the past month, has the COVID-19 pandemic affected your work or ability to work?’ and ‘In the past month, has the COVID-19 pandemic affected your school or college work?’. Each question was rated on a scale with response categories of 0 for not at all, 1 for slightly, 2 for moderately, 3 for very much and 4 for extremely. Each question was aggregated based on the two highest categories versus the rest (low disruption of COVID-19-related stressful events for categories 0–2 versus high disruption in COVID-19-related stressful events for categories 3–4). In addition, we created a severity index of 0–3 to indicate the number of COVID-19-related stressful events.

#### Outcome variable

Screening for serious mental illness was measured using the six-item K6 scale,^6^ with direct reference to COVID-19 pandemic in the instructions. Items included: ‘During the past 30 days, how often did you feel: 1. nervous; 2. hopeless; 3. restless or fidgety; 4. so depressed that nothing could cheer you up; 5. everything was an effort; 6. worthless?’. Each category was rated as one of following: 0, none of the time; 1, a little of the time; 2, some of the time; 3, most of the time; or 4, all of the time. Scores ranged from 0 to 24, with ≥13 indicating elevated risk of serious mental illness.^6^ Cronbach α was satisfactory (0.90).

#### Statistical analyses

A multivariate logistic regression with elevated risk of serious mental illness (K6 score  ≥ 13) as the outcome measure entered the following variables: demographics (age, gender, marital status, background illness, isolation) and three COVID-19-related stressful events. A second logistic regression also included an index for cumulative COVID-19-related stressful events (ranging from zero to three) instead of each single event, as in the former logistic regression. Each category in the index was compared with the reference group (no COVID-19-related stressful events). For each variable, we calculated the odds ratio and 95% confidence interval, using SPSS version 25 for Windows (IBM).

### Study 2

The above study included only a limited number of COVID-19-related stressful events, and so did not cover a number of major aspects of life. In our second study, we aimed to replicate the findings of study 1, using a different national sample and an expanded list of stressful events. We collected data as the pandemic's first wave in the UK plateaued and the four constituent countries moved out of lockdown.

In this second study, we considered an additional aspect of the mental health toll of the COVID-19 pandemic: ICD-11 adjustment disorder. Contrary to serious mental illness, a broad concept based on multiple constructs mainly drawing on depression and anxiety,^6^ the ICD-11 adjustment disorder is specifically associated with stress, providing a distinct mental disorder (adjustment disorder: code 6B43 in ICD-11) alongside a specific narrative description to provide and guide specific diagnosis.^7^

Based on study 1, we hypothesised that COVID-19-related stressful events will be associated with elevated risk of serious mental illness and probable ICD-11 adjustment disorder. Based on a dose–response model,^7^ we predicted that the more stressful events a person experiences, the higher the likelihood that they will exhibit elevated risk of serious mental illness and ICD-11 adjustment disorder.

#### Sampling

We conducted a sample of the UK population, using an internet panel (*N* = 1073), with random stratified sampling. Participants were recruited between 17 and 23 June 2020. The mean age of the participants was 54.36 years (s.d. 12.14, range 20–75), 55.1% were female (*n* = 591), 70.5% reported being in a committed relationship (*n* = 757) and 28.6% (*n* = 307) reported having a background medical condition (hypertension, diabetes, cardiovascular disease, chronic respiratory disease, chronic obstructive pulmonary disease and cancer). The response rate was 42%. See Table 1 for more information.

#### Measurements

We used similar measures as in study 1, with the following exceptions. COVID-19-related stressful events were measured by the International Adjustment Disorder Questionnaire (IADQ).^7^ The IADQ comprises two parts. First is a COVID-19-modified checklist of stressful events (stressors list) with eight events: financial problems (e.g. difficulty paying bills, being in debt); work problems (e.g. unemployment, redundancy, retirement, problems/conflicts with colleagues, change of job role); educational problems (e.g. difficulty with course work, deadline pressure); housing problems (e.g. stressful home move, difficulty finding a secure residence, lack of secure residence); relationship problems (e.g. break-up, separation or divorce, conflict with family or friends, intimacy problems); personal health problems (e.g. illness onset or deterioration, medication issues, injury or disability); a loved one's health problems (e.g. illness onset or deterioration, medication issues, injury or disability) and caregiving problems (e.g. emotional stress, time demands). Participants checked ‘yes’ for each event applicable to them, creating a cumulative score categorised as follows: 0, zero events; 1, one event; 2, two events; 3, three events and 4, four or more events (the number of participants reporting more than four events was 67 (6.1%)). The second IADQ component assesses adjustment disorder core symptoms, duration and functional impairment. The first six questions measure two symptoms clusters (‘preoccupation’ and ‘failure to adapt’). Three subsequent questions examine functional impairment in life domains (social, occupational (educational) and other domains of life). These nine questions were rated as follows: 0, not at all; 1, a little bit; 2, moderately; 3, quite a bit and 4, extremely. The tenth question assesses duration of symptoms (coded as 0 for no and 1 for yes).

The algorithm for a probable diagnosis of ICD-11 adjustment disorder requires the presence of a psychosocial stressor (score ≥1 on the IADQ stressor list), at least one preoccupation symptom rated ≥2, at least one failure-to-adapt symptom rated ≥2, and evidence of functional impairment rated ≥2.^7^ Cronbach α was satisfactory for IADQ (0.97).

#### Outcome variables

To limit the tautological risk of using the stressor list as both an independent and dependent variable, we computed a second, modified algorithm, which excludes the IADQ stressor list.

To use the second part of the IADQ (adjustment disorder scale) as an outcome measure, we viewed the COVID-19 pandemic as a global stressor, thus satisfying the criteria for exposure to a stressful event. We used a slightly modified algorithm to prevent a tautology in this study, utilising the stressor list as part as the dependent and independent variables (measuring exposure to COVID-19-related stressful events as the independent variable and as part of the dependent variable). The modified algorithm used in the study is identical to algorithm presented above excluding the IADQ stressor list.

The results section includes the prevalence of probable ICD-11 adjustment disorder among the UK population according to the IADQ original algorithm, and the prevalence of ICD-11 adjustment disorder when using the modified algorithm.

#### Serious mental illness

Screening for serious mental illness^6^ was measured in the same way as in study 1. Cronbach α was satisfactory for K6 (0.92).

#### Statistical analysis

A multivariate logistic regression used elevated risk of serious mental illness (K6 score ≥ 13) as the outcome measure, with the following variables entering the equation: demographics (age, gender, marital status, background illness, isolation) and exposure to COVID-19-related stressful event (with all events entered simultaneously). The second logistic regression was the same, except that we used an index for cumulative COVID-19-related stressful events (ranging from zero to eight) instead of the eight single events. Each category in the index was compared with the reference group (zero COVID-19-related stressful events). The same method was used, with probable ICD-11 adjustment disorder as the outcome variable (based on the modified algorithm). For each variable, we calculated the odds ratio and 95% confidence interval, and conducted a comparison of proportions, comparing the percentage of those with elevated risk of serious mental illness in each sample (independent sample comparison). Finally, we compared the models associated with elevated psychological distress with the modified algorithm for ICD-11 probable adjustment disorder, using a chi-square test.

To test the dose–response model for stress, we compared the association of each stressful event and the total events score with elevated risk of serious mental illness and modified algorithm for ICD-11 probable adjustment disorder, respectively, using Steiger's *z*-test.^8^ Data were analysed with SPSS version 25 for Windows (IBM).

## Results

For study 1, elevated risk of serious mental illness was found in 16.6% of the sample (*n* = 215). Elevated risk of serious mental illness was associated with disruption in social (odds ratio 2.59; 95% CI 1.81–3.71; *P* < 0.001) and occupational domains (odds ratio 1.53; 95% CI 1.06–2.21; *P* = 0.022). (Table 1). Elevated risk of serious mental illness was associated with cumulative COVID-19-related stressful events: odds ratio of 1.65 for one COVID-19-related stressful event (95% CI 1.03–2.64; *P* = 0.037), 2.43 for two COVID-19-related stressful events (95% CI 1.53–3.87; *P* < 0.001) and 5.56 for three COVID-19-related stressful events (95% CI 3.60–8.60; *P* < 0.001) (Table 2).

**Table 2:** Logistic regressions in the UK sample from March–April 2020, for the association of the study variables with serious mental illness (study 1; *N* = 1293)

Variables	Elevated risk of serious mental illness (K6 score ≥ 13)			
	Adjusted odds ratio (95% CI)	*P-*value	Adjusted odds ratio (95% CI)	*P*-value
Demographics				
Age, years	0.98 (0.96–0.99)	<0.001	0.98 (0.97–0.99)	<0.001
Gender, female	1.38 (1.00–1.90)	0.049	1.39 (1.01–1.91)	0.045
Being in a committed relationship, yes	1.15 (0.82–1.61)	0.425	1.19 (0.85–1.67)	0.319
Being in risk group for COVID-19, yes	1.30 (0.90–1.89)	0.166	1.29 (0.90–1.87)	0.186
Lockdown				
Currently in isolation, yes	1.69 (1.21–2.34)	0.002	1.72 (1.24–2.40)	0.001
Disrupted COVID-19-related stressful events				
High disruption in social domain, yes	2.59 (1.81–3.71)	<0.001		
High disruption in occupational domain, yes	1.53 (1.06–2.21)	0.022		
High disruption in educational domain, yes	1.37 (0.95–1.98)	0.092		
Index of disrupted COVID-19-related stressful events				
No disruption in COVID-19-related stressful events				
One disruption in COVID-19-related stressful events			1.65 (1.03–2.64)	0.037
Two disruptions in COVID-19-related stressful events			2.43 (1.53–3.87)	<0.001
Three disruptions in COVID-19-related stressful events			5.56 (3.60–8.60)	<0.001

The results of study 2 showed that elevated risk of serious mental illness was found in 14.3% of the sample (*n* = 153). The proportion comparison between the two samples indicated that elevated risk of serious mental illness was not significant (difference of 2.3%; 95% CI –0.64% to 5.20%; *χ*^2^ = 2.360; d.f. 1; *P* = 0.125).

Probable ICD-11 adjustment disorder according to the IADQ algorithm was found among 15.9% of the sample (*n* = 171), and probable ICD-11 adjustment disorder according to the IADQ modified algorithm was found among 17.8% of the sample (*n* = 191).

Elevated risk of serious mental illness was significantly associated with housing problems (odds ratio 2.71; 95% CI 1.39–5.30; *P* = 0.003), the participant's own health problems (odds ratio 3.43; 95% CI 2.20–5.30; *P* < 0.001) and caregiving problems (odds ratio 1.83; 95% CI 1.14–2.94; *P* = 0.013). Elevated risk of serious mental illness was significantly associated with cumulative COVID-19-related stressful events compared with those participants reporting no COVID-19-related stressful events. These results were significant for those who had experienced at least one COVID-19-related stressful event (odds ratio 2.19; 95% CI 1.15–4.15; *P* = 0.017), with an increased likelihood of elevated risk of serious mental illness for those reporting experiencing four or more COVID-19-related stressful events (odds ratio 12.41; 95% CI 7.05–21.84; *P* < 0.001). In addition, being younger was associated with elevated risk of serious mental illness for both measures. For more information, see Table 3. These results echoed the results of study 1.

**Table 3:** Logistic regressions in the UK sample from June 2020, for the association of the study variables with serious mental illness (study 2; *N* = 1073)

Variables	Elevated risk of serious mental illness (K6 score ≥ 13)			
	Adjusted odds ratio (95% CI)	*P*-value	Adjusted odds ratio (95% CI)	*P*-value
Demographics				
Age, years	0.96 (0.95–0.98)	<0.001	0.97 (0.96–0.99)	<0.001
Gender, Female	1.45 (0.97–2.15)	0.069	1.33 (0.90–1.97)	0.146
Being in a committed relationship, yes	1.39 (0.92–2.09)	0.114	1.42 (0.95–2.13)	0.090
Being in risk group for COVID-19, yes	1.14 (0.73–1.77)	0.575	1.42 (0.94–2.16)	0.098
Lockdown				
Currently in isolation, yes	1.62 (0.96–2.73)	0.068	1.54 (0.91–2.59)	0.107
COVID-19-related stressful events, yes				
Financial problems	1.45 (0.92–2.30)	0.113		
Work problems	0.86 (0.52–1.45)	0.581		
Educational problems	0.82 (0.36–1.89)	0.643		
Housing problems	2.71 (1.39–5.30)	0.003		
Relationship problems	1.42 (0.86–2.33)	0.174		
Personal health problems	3.43 (2.20–5.33)	<0.001		
A loved one's health problems	1.29 (0.82–2.04)	0.269		
Caregiving problems	1.83 (1.14–2.94)	0.013		
Index of COVID-19-related stressful events				
No events				
One event			2.19 (1.15–4.15)	0.017
Two events			3.68 (2.03–6.65)	<0.001
Three events			6.98 (3.78–12.90)	<0.001
Four or more events			12.41 (7.05–21.84)	<0.001

A similar pattern emerged with regard to probable ICD-11 adjustment disorder according to the modified algorithm. Probable ICD-11 adjustment disorder was significantly associated with the participant's own health problems (odds ratio 2.07; 95% CI 1.38–3.10; *P* < 0.001), the participant's loved one's health problems (odds ratio 1.85; 95% CI 1.22–2.80; *P* = 0.004) and caregiving problems (odds ratio 2.59; 95% CI 1.68–3.99; *P* < 0.001). Using the modified algorithm, probable ICD-11 adjustment disorder was significantly associated with cumulative COVID-19-related stressful events compared with those participants reporting no COVID-19-related stressful events. These results were significant for those who had experienced at least one COVID-19-related stressful event (odds ratio 2.45; 95% CI 1.27–4.72; *P* = 0.007), with an increased likelihood of probable ICD-11 adjustment disorder for those reporting experiencing four or more COVID-19-related stressful events (odds ratio 17.81; 95% CI 9.92–31.97; *P* < 0.001). In addition, being younger and currently being in isolation were associated with elevated serious mental illness for both measures. For more information, see Table 4.

**Table 4:** Logistic regressions in the UK sample from June 2020, for the association of the study variables with ICD-11 adjustment disorder (study 2; *N* = 1073)

Variables	ICD-11 probable adjustment disorder (modified algorithm)			
	Adjusted odds ratio (95% CI)	*P*-value	Adjusted odds ratio (95% CI)	*P*-value
Demographics				
Age, years	0.90 (0.97–1.00)	0.080	0.99 (0.099–1.02)	0.092
Gender, female	1.13 (0.79–1.62)	0.510	1.01 (0.70–1.49)	0.963
Being in a committed relationship, yes	1.43 (0.98–2.09)	0.065	1.21 (0.82–1.77)	0.336
Being in risk group for COVID-19, yes	0.90 (0.59–1.37)	0.631	0.96 (0.64–1.43)	0.825
Lockdown				
Currently in isolation, yes	1.89 (1.16–3.06)	0.010	1.90 (1.16–3.10)	0.011
COVID-19-related stressful events				
Financial problems, yes	1.48 (0.96–2.27)	0.076		
Work problems, yes	1.37 (0.86–2.19)	0.187		
Educational problems, yes	1.60 (0.71–3.59)	0.253		
Housing problems, yes	1.39 (0.71–2.73)	0.335		
Relationship problems, yes	1.32 (0.82–2.11)	0.249		
Personal health problems, yes	2.07 (1.38–3.10)	<0.001		
A loved one's health problems, yes	1.85 (1.22–2.80)	0.004		
Caregiving problems, yes	2.59 (1.68–3.99)	<0.001		
Index of COVID-19-related stressful events				
No events				
One event			2.45 (1.27–4.72)	0.007
Two events			9.56 (5.44–16.80)	<0.001
Three events			16.64 (9.08–30.51)	<0.001
Four or more events			17.81 (9.92–31.97)	<0.001

In addition, we tested the models for association with the aforementioned (independent) variables examining the elevated risk of serious mental illness and the modified algorithm for probable ICD-11 adjustment disorder. The –2log likelihood in the first model (Table 3) associated with elevated risk of serious mental illness was 723.272. This compares with 828.809 for the –2log likelihood for the model for probable ICD-11 adjustment disorder (Table 4). The results of the chi-square test were significant at *P* < 0.005. Hence, there was better model fit for the elevated risk of serious mental illness.

Finally, we tested the dose–response model for elevated risk for serious mental illness. A Steiger's *z*-score of 1.609 indicates that the dose–response model was not significant for elevated risk of serious mental illness. However, the modified algorithm for probable ICD-11 adjustment disorder showed a significant Steiger's *z*-score of 2.375, indicating a dose–response association.

## Discussion

The results of study 1 showed that elevated risk of serious mental illness was associated with disruption in social and occupational COVID-19-related stressful events, but not with those more educational aspects. As more COVID-19-related stressful events were generated, the risk of serious mental illness increased, suggesting the risk of overwhelming an individual's psychological capacities during times of enhanced stress.^9^ These COVID-19-related stressful events have important implications for daily functioning and preserving psychological well-being. In terms of social relationships, an inability to seek help outside the home means that COVID-19 has been associated with increased domestic problems.^10^ In the context of the UK, it is estimated that the current gross domestic product will shrink by 9% and around 7.6 million jobs will be lost. Roughly 24% of the UK labour force are at risk because of COVID-19-related lockdowns.^11^ Those with the lowest income are the most vulnerable.^12^ This may suggest that the most vulnerable groups are prone to exhibit elevated serious mental illness when compared with less vulnerable groups.

The results pattern of study 2 were similar to those in study 1, albeit with different COVID-19-related stressful events. Elevated serious mental illness and probable ICD-11 adjustment disorder were associated with participants’ own health and caregiving problems and, to a lesser extent, housing problems and the health problems of a loved one. In addition, the model associated with elevated serious mental illness was found to be more robust than the model associated with the modified ICD-11 adjustment disorder. This may be because the K6 scale is capturing more aspects of anxiety and depression.

Cumulative stressful events take their medical toll. This has been found in the association between cumulative stress and disrupted hypothalamic-pituitary-adrenocortical axis functioning,^13^ lower oxytocin level in pregnant women^14^ and Takotsubo cardiomyopathy.^15^ This adds to the known detrimental effect of psychological stress on health (e.g. immunoregulatory balance) and the association with inflammatory disease,^16^ cardio vascular disease,^17^ immune system^18^ and immune inflammatory reactions.^19^ Psychological stress also takes an economic toll directly and indirectly, and increases the burden on the health system.^20^

The results of study 2 support a dose–response model of stress and post-traumatic stress disorder;^21^ the more COVID-19-related stressful events were experienced by the participants, the more the mental toll, evidenced in an increased likelihood of probable ICD-11 adjustment disorder. The dose–response association of cumulative disruptions in COVID-19-related stressful events or cumulative COVID-19-related stressful events on probable ICD-11 adjustment disorder echoes previous findings.^22,23^

From a clinical point of view, these disruptions and COVID-19-related stressful events are important, as they can readily lead to an excessive burden on the general population. Clinicians may expect to see more referrals based on resource loss and any ineffective coping associated with this. Another consequence of the COVID-19 pandemic is that populations generally at lower risk of mental disorder before COVID-19 may become at risk because of the disruption to their everyday lives.^24–26^ Our findings suggest that clinicians should be aware of disruption in COVID-19-related stressful events as a source of serious mental illness, especially when there is risk of a ‘storm’ that overwhelms an individual's capacity to cope. Although the association between a medical cytokine storm and health outcomes from COVID-19 disease^27,28^ has recently been questioned,^29^ COVID-19 is a global stressful event, with salient psychosocial consequence.

### General limitations and conclusions

We recognise several study limitations. Our study was cross-sectional and, although we recruited respondents from a wide range of ages, our responses were self-reported. In addition, we used two unrelated samples recruited at different time points. This is a weakness when compared with a longitudinal study. We also report relatively low response rates (37% and 42%, respectively), although these response rates are similar to other large internet surveys.^30^ We had no information on past psychological conditions. We did not consider additional psychological consequences of anxiety, such as stereotyping and prejudice, reported during the SARS^31^ and COVID-19 pandemics.^32,33^ Another potential bias is the possibility that people who are distressed about COVID-19 may be more likely to fill out a survey, thus inflating the strength of the relationship.

The cost of psychological stress is known to be associated with medical conditions and illnesses,^34^ and increased risk for mortality.^21^ Psychiatrists should be aware that certain COVID-19-related stressful events can lead to serious psychological problems.^24^ They need to pay particular attention to patients who report cumulative COVID-19-related stressful events, and consider them for mental health assessment and treatment.

In sum, to our knowledge, this is first study to empirically examine the association between COVID-19-related stressful events and cumulative COVID-19-related stressful events, with an elevated risk of serious mental illness at two different time points. Moreover, we report a novel association between single COVID-19-related stressful events and cumulative COVID-19-related stressful events with probable ICD-11 adjustment disorder.

Future studies should take a longitudinal approach, and use biological markers of stress to gain a better insight into the health costs of experiencing specific and cumulative COVID-19-related stressful events.

## Data Availability

The data that support the findings of this study are available from the corresponding author, M.B.-E., upon reasonable request..

## Supplementary material

For supplementary material accompanying this paper visit https://doi.org/10.1192/bjo.2020.158. click here to view supplementary material
